# Association of alcohol consumption with the incidence of proteinuria and chronic kidney disease: a retrospective cohort study in Japan

**DOI:** 10.1186/s12937-022-00785-x

**Published:** 2022-05-14

**Authors:** Akio Tanaka, Makoto Yamaguchi, Takuji Ishimoto, Takayuki Katsuno, Hironobu Nobata, Shiho Iwagaitsu, Hirokazu Sugiyama, Hiroshi Kinashi, Shogo Banno, Takahiro Imaizumi, Masahiko Ando, Yoko Kubo, Yasuhiko Ito

**Affiliations:** 1 Department of Nephrology and Rheumatology, Aichi Medical University, Nagakute, Japan; 2 Department of Pharmacy, Daido Hospital, Nagoya, Japan; 3 Data Coordinating Center, Department of Advanced Medicine, Nagoya University Hospital, Nagoya, Japan; 4 Department of Preventive Medicine, Nagoya University Graduate School of Medicine, Nagoya, Japan

**Keywords:** Alcohol, Chronic kidney disease, Proteinuria, Sex difference, Retrospective cohort study

## Abstract

**Background:**

The difference in the clinical impact of alcohol consumption on kidney function based on sex remains to be elucidated. This study aimed to assess the association between the dose of alcohol consumption and the incidence of proteinuria and chronic kidney disease stratified by sex.

**Methods:**

This retrospective cohort study included 26,788 workers (19,702 men and 7086 women) with normal renal function (estimated glomerular filtration rate ≥ 60 mL/min/1.73 m^2^) at annual health examinations between January 2010 and March 2015 in Japan. The main exposure was alcohol consumption. The primary outcomes were the incidence of proteinuria (dipstick urinary protein ≥ 1) and incidence of low estimated glomerular filtration rate (eGFR; rate < 60 mL/min per 1.73 m^2^; decreased from the baseline eGFR by 25%).

**Results:**

During a median observational period of 4 years (interquartile range: 2–6), 1993 (10.1%) men and 462 (6.5%) women developed proteinuria, whereas 667 (3.4%) men and 255 (3.6%) women developed low eGFR. After adjustment for clinically relevant factors using a Cox proportional hazards model, alcohol consumption of ≥ 46 g/day in females was significantly associated with the incidence of proteinuria (hazard ratio, 1.57; 95% confidence interval, 1.10–2.26) and low eGFR (hazard ratio, 1.62; 95% confidence interval, 1.04–2.53). However, no significant association between alcohol consumption and primary outcomes was observed in men.

**Conclusions:**

In conclusion, daily higher alcohol consumption was significantly associated with a higher incidence of proteinuria and low eGFR among women. Women might be prone to high alcohol consumption with kidney dysfunction.

**Supplementary Information:**

The online version contains supplementary material available at 10.1186/s12937-022-00785-x.

## Background

Chronic kidney disease (CKD) is a risk factor for cardiovascular disease, development of end-stage renal disease (ESRD), and all-cause mortality. Furthermore, CKD is considered an important global health problem owing to its socioeconomic burden [1]. Therefore, it is essential to identify the modifiable risk factors for the development of CKD and effective strategies for preventing it.

Alcohol consumption is a lifestyle-related factor associated with mortality. Although excess alcohol intake has been associated with increased mortality [2], moderate drinkers generally have lower mortality rates than non-drinkers or heavy drinkers in population-based observational studies, demonstrating J-shaped associations [3–7]. This may be because excess alcohol consumption may cause several adverse effects, including liver disease, heart failure, increased cancer risk, neurologic complications, and unintentional injuries. Moderate alcohol consumption most likely reduces the risk of coronary heart disease through its effects on insulin sensitivity, thrombotic activity, and inflammation, leading to reduction in cardiovascular mortality [8, 9]. As for the influence of alcohol on kidney function, although several population-based cohort studies have evaluated the relationship between the amount of alcohol consumption and incidence of CKD, these results have been inconsistent; an inverse association [10–13], a positive association [14–17], and a J-shaped association [18–23] between alcohol intake and incidence of CKD have been reported in longitudinal studies involving the general population. Furthermore, although several studies have evaluated the difference in the clinical impact of alcohol consumption on kidney function based on sex [12, 17, 22, 23], the results have been inconsistent.

This retrospective cohort study of 26,788 individuals from the general population in Japan aimed to assess the association between the dose of alcohol consumption and the incidence of proteinuria and CKD stratified by sex. The results of this study may provide important implications for the clinical effect of alcohol consumption as a key lifestyle-related factor associated with the incidence of CKD.

## Methods

### Study population

**Fig. 1:**
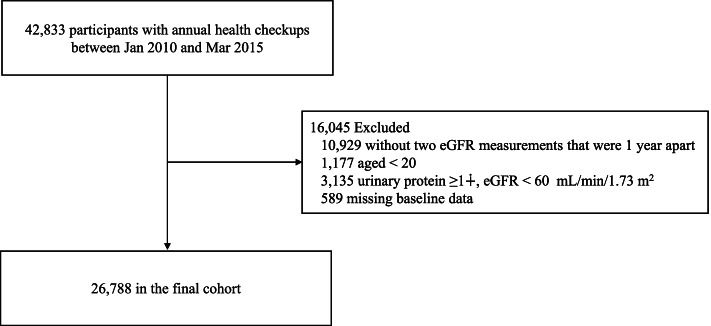
Flow diagram of participant selection

Overall, 42,833 workers from the Daido corporation group, aged 18–80 years, were eligible for inclusion in the present retrospective cohort study. These individuals had visited the Daido hospital for their annual health checkups for health insurance during the entry period between January 2010 and March 2015. Of 38,521 (89.9%) participants aged ≥ 20 years with no proteinuria (dipstick urinary protein ≤ ±) and an estimated glomerular filtration rate (eGFR) of ≥ 60 mL/min/1.73 m2; 589 participants with missing data on alcohol consumption at baseline at their first visit, and 10,929 without two eGFR measurements with a 1-year interval during the observational period between January 2010 and December 2018, were excluded. Finally, 26,788 participants (19,702 men and 7086 women) with normal renal function (no proteinuria and eGFR of ≥ 60 mL/min/1.73 m2) were included in the analysis (Fig. 1).

To calculate eGFR, the following Japanese equation was used: eGFR (mL/min/1.73 m^2^) = 194 × age (years)^−0.287^ × serum creatinine (mg/dL)^−1.094^ × 0.739, if female [24].

The protocol of the present study was approved by the Daido Hospital and Aichi Medical University (no. 2020–8). An opt-out approach was adopted regarding informed patient consent, according to the Japanese Ethical Guidelines for Medical and Health Research involving human subjects.

### Measurements

#### Predictors and covariates

Because of the retrospective nature of the present study, the sample size was determined by the number of employees of the Daido corporation who visited Daido Hospital for their annual health checkup during the entry period. For each participant, baseline data were collected at the first visit for annual heath checkups between January 2010 and March 2015. These baseline data, collected using self-administered questionnaires, included that for age, sex, body mass index (BMI), systolic and diastolic blood pressure, serum levels of total cholesterol, triglyceride, hemoglobin A1c (NGSP), eGFR, and urinary protein level in the random void urine sample assessed by the dipstick method: negative (-), ± , 1 + , 2 + , or 3 + , lifestyle (alcohol consumption and smoking status [non-smoker, past smoker, and current smoker]) and current treatment for comorbidities at baseline (hypertension, hyperlipidemia, diabetes mellitus, and cardiovascular disease). The main exposure of the present study was the amount of alcohol consumed.

Alcohol consumption was ascertained by asking the following two questions: “How often do you drink alcoholic beverages: every day, 5–6 days/week, 3–4 days/week, 1–2 days/week, a few times/month, or seldom/can’t” and “How many alcoholic beverages do you drink on the days you drink: no consumption, < 1 go per day; 1 go per day; 2–3 go per day; or ≥ 3 go per day?” In Japan, the standard serving size of an alcoholic beverage, “1 go,” is equivalent to approximately 180 mL of Japanese sake, or 23 g of ethanol, and is the most common unit for measuring the amount of alcohol consumed. We assigned a score to each category of alcohol consumption frequency as follows: 7 for every day, 5.5 for 5–6 days/week, 3.5 for 3–4 days/week, 1.5 for 1–2 days/week, 0.5 for a few times/month, and 0 for seldom/can’t, according to a previous study [25]. Finally, the weekly ethanol equivalent intake was estimated by multiplying the amount of ethanol by the frequency score; the daily ethanol equivalent intake was calculated by dividing these estimates, and was categorized as no consumption, < 23 g/day, 23–46 g/day, or ≥ 46 g /day [26].

Follow-up data regarding annual urinary protein and eGFR levels were also collected.

#### Outcomes

The primary outcomes of interest were 1) incident proteinuria, defined as a urinary protein ≥ 1 + , as assessed by the dipstick test and 2) incident low eGFR, defined as two eGFR levels < 60 mL/min/1.73 m^2^ and a 25% decrease from baseline eGFR [27].

The observational period for evaluating the incidence of proteinuria was designated as the time from the baseline visit to the incidence of proteinuria or the last measurement of urinary protein before the end of December 2018, whichever came first. The observational period for evaluating the incidence of low eGFR was designated as the time from the baseline visit to the first development of eGFR levels of < 60 mL/min/1.73 m^2^ and a 25% decrease from baseline eGFR or the last measurement of eGFR before the end of December 2018, whichever came first.

### Statistical analysis

Baseline characteristics according to alcohol consumption categories in men and women were compared using the Kruskal–Wallis test or Pearson's chi-square test, as appropriate. For time to event analyses, the associations of alcohol consumption with outcomes were assessed using the Kaplan–Meier method, log-rank tests, and Cox proportional hazards models; patients were followed up until censored at the date of the last check-up or December 2018 for incident proteinuria and low eGFR, respectively; person-years were also calculated. Multivariable models were incrementally adjusted for the following clinically relevant factors on the basis of prior consideration of their associations with worsening kidney function, according to previous studies [28, 29]; model 1 was adjusted for age (year), sex, and baseline eGFR (mL/min/1.73 m^2^), model 2 additionally included the BMI (kg/m^2^) and smoking status (never/former and current smokers), and model 3 additionally accounted for current treatment for comorbidities (hypertension, dyslipidemia, diabetes mellitus, and cardiovascular disease). The proportionality assumption was tested by plotting log [2log (survival rate)] against log (survival time). All associations were examined using unadjusted and multivariable-adjusted models. The alcohol consumption trend associated with each outcome was examined statistically by scoring no as 0 and < 23 g, 23–46 g, and ≥ 46 g/day as 1, 2, and 3, respectively; the resulting scores were then included in the regression model. For detection of a possible interaction between alcohol consumption and incidence of low eGFR, we included sex and alcohol consumption as variables in the Cox model, and a significant interaction by sex was observed (*P* < 0.001). This suggested that the effects of alcohol on the kidney may differ between the sexes; subsequently, all analyses were performed separately for males and females. We also performed a multiple imputation analysis as a sensitivity analysis for missing data regarding alcohol consumption (0.2%) and clinical outcome (0.1%).

Continuous variables were expressed as medians and interquartile ranges and categorical variables as numbers and proportions. Statistical significance was set at a two-tailed *P-*value of < 0.05. Statistical analyses were performed using Stata software version 15.0 (StataCorp LP, College Station, TX, USA) and JMP software version 14.0.0 (SAS Institute, Cary, NC, USA).

## Results

### Baseline characteristics

The baseline characteristics of 19,902 men and 7086 women according to alcohol consumption categories are listed in Tables 1.

**Table 1:** Baseline characteristics of 19,902 males and 7086 females

	**Male**				**Female**			
	**Daily alcohol consumption categories (g of alcohol)**				**Daily alcohol consumption categories (g of alcohol)**			
	**No**	** < 23 g**	**23–46**	** ≥ 46**	**No**	** < 23 g**	**23–46**	** ≥ 46**
**Number**	7352	6337	4252	1761	4017	1952	755	362
Age (years) *	39 (31–49)	40 (32–50)	47 (39–57)	48 (40–57)	42 (35–51)	42 (35–50)	46 (39–55)	47 (39–55)
Smoking, *n* (%) *								
**Never**	2793 (38.0)	2109 (33.3)	757 (17.8)	195 (11.1)	3258 (81.1)	1500 (76.8)	486 (64.4)	239 (66.0)
**Former**	1714 (23.3)	1862 (29.4)	1580 (37.2)	647 (36.7)	404 (10.1)	278 (14.2)	136 (18.0)	64 (17.7)
**Current**	2803 (38.1)	2366 (37.3)	1915 (45.0)	919 (52.2)	301 (7.5)	170 (8.7)	127 (16.8)	56 (15.5)
**Body mass index, kg/m**^2^*	23.3 (21.1–25.9)	23.1 (21.3–25.3)	23.3 (21.6–25.3)	23.3 (21.5–25.3)	21.1 (19.3–23.6)	21.1 (19.3–23.4)	21.6 (19.8–24.1)	21.9 (19.8–24.3)
Systolic BP, mmHg *	119 (109–130)	119 (110–130)	120 (110–132)	121 (111–132)	112 (102–125)	112 (102–124)	112 (102–125)	115 (104–126)
Diastolic BP, mmHg *	72 (65–81)	73 (66–81)	75 (67–84)	76 (67–84)	68 (61–76)	68 (61–76)	68 (61–77)	70 (61–77)
**Uric acid, mg/dL**	5.8 (4.9–6.7)	5.8 (5.0–6.7)	5.9 (5.0–6.7)	5.9 (5.0–6.7)	4.5 (3.8–5.5)	4.6 (3.8–5.5)	4.4 (3.7–5.4)	4.5 (3.8–5.3)
Total cholesterol, mg/dL *	196 (173–222)	197 (175–221)	203 (182–226)	205 (183–227)	199 (178–225)	199 (176–223)	202 (179–228)	202 (180–230)
Triglyceride, mg/dL *	96 (66–142)	96 (66–142)	102 (71–152)	109 (74–171)	70 (51–98)	68 (51–95)	76 (59–105)	84 (61–127)
Hemoglobin A1c, % *	5.4 (5.2–5.7)	5.4 (5.2–5.6)	5.4 (5.2–5.7)	5.4 (5.2–5.7)	5.4 (5.2–5.7)	5.4 (5.2–5.6)	5.4 (5.2–5.6)	5.4 (5.2–5.7)
**eGFR, mL/min/1.73 m**^2^*	87 (77–97)	86 (76–96)	83 (75–94)	85 (76–96)	75 (68–84)	76 (68–84)	73 (67–82)	76 (69–84)
**Current treatments**								
Hypertension, *n* (%) *	426 (5.8)	500 (7.9)	611 (14.4)	306 (17.4)	188 (4.7)	95 (4.9)	77 (10.2)	42 (11.6)
Dyslipidemia, *n* (%) *	242 (3.3)	275 (4.3)	192 (4.5)	84 (4.8)	147 (3.7)	63 (3.2)	43 (5.7)	26 (7.2)
Diabetes mellitus, *n* (%) *	210 (2.9)	176 (2.8)	146 (3.4)	69 (3.9)	50 (1.2)	19 (1.0)	12 (1.6)	8 (2.2)
CVD, *n* (%) *	37 (0.5)	29 (0.5)	31 (0.7)	8 (0.5)	11 (0.3)	3 (0.2)	3 (0.4)	1 (0.3)

Continuous data are presented as a median (interquartile range) and categorical data are expressed as a number (proportion)

Abbreviations; *BP* blood pressure, *eGFR* estimated glomerular filtration rate, *CVD* cardiovascular disease;

^*^p < 0.05 among 4 categories of alcohol consumption

Among male participants, non-drinkers [*n* = 7352 (37.3%)] were the most common, followed by those with consumption levels of < 23 g/day [*n* = 6337 (32.2%)], 23–46 g/day [*n* = 4252 (21.6%)], and ≥ 46 g/day [*n* = 1761 (8.9%)] (Table 1). Individuals with a higher alcohol consumption tended to be older, smokers, dyslipidemic, receiving treatment for hypertension, and diabetic.

Among women, non-drinkers were the most common [*n* = 4017 (56.7%)], which was at a higher proportion than that in men, followed by those consuming < 23 g/day [*n* = 1952 (27.5%)], 23–46 g/day [*n* = 755 (10.7%)], and ≥ 46 g/day [*n* = 362 (5.1%)] (Table 2). Similar trends were observed for age, smoking, dyslipidemia, and treatment for hypertension. The amount of daily alcohol consumption was higher in males than in females (7 (0–28) g and 0 (0–8) g in males and females, respectively [*P* < 0.001]).

**Table 2:** Alcohol consumption and the incidence of proteinuria in males and females

	**Daily alcohol consumption categories (g of alcohol)**				***P*-trend**
	**No**	** < 23 g**	**23–46**	** ≥ 46**	
**Males**					
Incidence of proteinuria, *n* (%)	744 (10.1)	612 (9.7)	434 (10.2)	203 (11.5)	
IR per 1000PY	26.7	24.7	25.3	29.8	
Hazard ratio (95% CI)					
Unadjusted model	1.0 (reference)	0.92 (0.83–1.02)	0.93 (0.83–1.05)	1.10 (0.94–1.28)	.852
Adjusted model 1	1.0 (reference)	0.94 (0.84–1.04)	0.99 (0.87–1.11)	1.14 (0.98–1.34)	.312
Adjusted model 2	1.0 (reference)	0.97 (0.87–1.08)	1.00 (0.89–1.13)	1.17 (0.99–1.37)	.196
Adjusted model 3	1.0 (reference)	0.97 (0.87–1.08)	0.99 (0.88–1.12)	1.14 (0.97–1.34)	.318
**Females**					
Incidence of proteinuria, *n* (%)	257 (6.4)	126 (6.5)	44 (5.8)	35 (9.7)	
IR per 1000PY	18.0	18.7	15.4	26.0	
Hazard ratio (95% CI)					
Unadjusted model	1.0 (reference)	1.03 (0.84–1.28)	0.86 (0.63–1.19)	1.44 (1.01–2.05) *	.369
Adjusted model 1	1.0 (reference)	1.04 (0.84–1.29)	0.97 (0.71–1.34)	1.66 (1.16–2.37) *	.077
Adjusted model 2	1.0 (reference)	1.04 (0.84–1.29)	0.93 (0.67–1.29)	1.59 (1.10–2.28) *	.140
Adjusted model 3	1.0 (reference)	1.04 (0.84–1.29)	0.93 (0.67–1.30)	1.57 (1.10–2.26) *	.144

*PY* person-years, *CI* confidence interval

Multivariate model 1 adjusted for age (years) and eGFR (mL/min/1.73 m^2^) at baseline

Model 2 adjusted for the covariates in model 1, body mass index (BMI) (kg/m^2^), and smoking status (never/former and current smokers)

Model 3 adjusted for the covariates in model 2, and current treatment for comorbidities (hypertension, dyslipidemia, diabetes mellitus, and cardiovascular disease)

^*^P < .05

P-trend was derived from general linear models by treating alcohol consumption as a continuous linear term

### Amount of alcohol consumption and risk of incident proteinuria

**Fig. 2:**
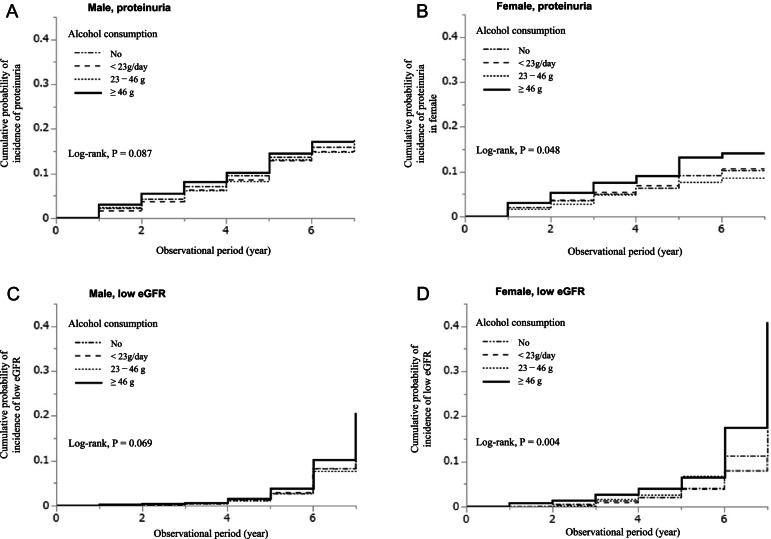
Cumulative probability based on alcohol consumption. The cumulative probability of incidence of proteinuria (≥ 1 +) in males (**A**) and females (**B**) and of low eGFR (eGFR < 60 mL/min/1.73 m^2^ and a 25% decrease) in males (**C**) and females (**D**)

During an observational period of 4 (3–6) years, 1993 (10.1%) males developed proteinuria (≥ 1 +) (Table 2). With respect to frequency of alcohol consumption, the incidence rates of proteinuria were 26.7, 24.7, 25.3, and 29.8 per 1000 person-years in the “no,” “ < 23 g,” “23–46 g,” and “ ≥ 46 g” consumption groups, respectively. The cumulative probability of incidence of proteinuria was comparable between the categories of alcohol consumption (*P* = 0.087) (Fig. 2A). Both unadjusted and multivariable Cox proportional hazards models showed no significant association between alcohol consumption and the incidence of proteinuria (Table 2).

Among women, during the observational period of 4 (2–5) years, 462 participants (6.5%) developed proteinuria (≥ 1 +) (Table 2). With respect to frequency of alcohol consumption, the incidence rates of proteinuria were 18.0, 18.7, 15.4, and 26.0 per 1000 person-years in the “no,” “ < 23 g,” “23–46 g,” and “ ≥ 46 g” consumption groups, respectively. The cumulative incidence of proteinuria in females was significantly higher in the > 46 g/day alcohol consumption group (log rank, *P* = 0.048) (Fig. 2B). Unadjusted Cox proportional hazards models showed that alcohol consumption of ≥ 46 g was significantly associated with the incidence of proteinuria [hazard ratios (95% confidence interval): non-drinker, 1.00 (reference), < 23 g, 1.03 (0.84–1.28), 23–46 g, 0.86 (0.63–1.19), and ≥ 46 g, 1.44 (1.01–2.05), respectively] (Table 2). After adjusting for clinically relevant factors, ≥ 46 g of alcohol consumption was identified as a significant predictor of the incidence of proteinuria [non-drinker, 1.00 (reference), < 23 g, 1.04 (0.84–1.29), 23–46 g, 0.93 (0.67–1.30), and ≥ 46 g, 1.57 (1.10–2.26), respectively] (model 3, Table 2).

### Amount of alcohol consumption and risk of incident low eGFR

A total of 719 men (3.5%) developed CKD during the observation period (Table 3). With respect to the categories of alcohol consumption, the incidence rates of low eGFR were 7.9, 8.0, 8.4, and 11.5 per 1000 person-years in the “no,” “ < 23 g,” “23–46 g,” and “ ≥ 46 g” groups, respectively. The cumulative probability of the incidence of low eGFR was comparable between the categories of alcohol consumption (*P* = 0.069) (Fig. 2C). Although the unadjusted Cox proportional hazards models showed that ≥ 46 g of alcohol consumption was associated with the incidence of low eGFR, the multivariable models showed that in males, the incidence of low eGFR was comparable between different alcohol consumption groups (Table 3).

**Table 3:** Alcohol consumption and the incidence of low eGFR in males and females

	**Daily alcohol consumption categories (g of alcohol)**				***P-* trend**
	**No**	** < 23 g**	**23–46**	** ≥ 46**	
**Males**					
Incidence of CKD, *n* (%)	228 (3.1)	206 (3.3)	150 (3.5)	83 (4.7)	
IR per 1000PY	7.9	8.0	8.4	11.5	
Hazard ratio (95% CI)					
Unadjusted model	1.0 (reference)	0.99 (0.83–1.21)	0.97 (0.79–1.20)	1.32 (1.03–1.70) *	.172
Adjusted model 1	1.0 (reference)	1.02 (0.84–1.23)	0.91 (0.73–1.12)	0.95 (0.73–1.24)	.432
Adjusted model 2	1.0 (reference)	1.03 (0.85–1.24)	0.91 (0.73–1.12)	0.96 (0.73–1.25)	.454
Adjusted model 3	1.0 (reference)	1.03 (0.85–1.24)	0.90 (0.73–1.11)	0.92 (0.71–1.20)	.342
**Females**					
Incidence of CKD, *n* (%)	120 (3.0)	69 (3.5)	38 (5.0)	28 (7.7)	
IR per 1000PY	8.3	10.0	13.0	20.1	
Hazard ratio (95% CI)					
Unadjusted model	1.0 (reference)	1.23 (0.91–1.65)	1.43 (0.99–2.06)	2.22 (1.47–3.36) *	< .001
Adjusted model 1	1.0 (reference)	1.23 (0.91–1.65)	1.21 (0.83–1.76) *	1.56 (1.01–2.39) *	.039
Adjusted model 2	1.0 (reference)	1.22 (0.91–1.65)	1.19 (0.81–1.76)	1.65 (1.06–2.56) *	.030
Adjusted model 3	1.0 (reference)	1.23 (0.91–1.65)	1.21 (0.83–1.79)	1.62 (1.04–2.53) *	.039

*PY* person-years, *CI* confidence interval

Multivariate model 1 adjusted for age (years) and eGFR (mL/min/1.73 m^2^) at baseline

Model 2 adjusted for the covariates in model 1, body mass index (BMI) (kg/m^2^), and smoking status (never/former and current smokers)

Model 3 adjusted for the covariates in model 2, and current treatment for comorbidities (hypertension, dyslipidemia, diabetes mellitus, and cardiovascular disease)

^*^P < .05

P-trend was derived from general linear models by treating alcohol consumption as a continuous linear term

Incidence of low eGFR was observed in 264 women (3.6%) during the observational period (Table 3). In contrast to men, the incidence rates of low eGFR were significantly higher in the ≥ 46 g group (Table 3); the incidence rates of low eGFR were 8.3, 10.0, 13.0, and 20.1 per 1000 person-years in the in the “no,” “ < 23 g,” “23–46 g,” and “ ≥ 46 g” groups, respectively. The cumulative probabilities of the incidence of low eGFR were significantly higher in the ≥ 46 g group (*P* = 0.004) (Fig. 2D).

The unadjusted Cox proportional hazards models showed that ≥ 46 g of alcohol consumption was significantly associated with the incidence of low eGFR [hazard ratios (95% confidence interval); non-drinker, 1.00 (reference); < 23 g, 1.23 (0.91–1.65); 23–46 g, 1.43 (0.99–2.06); and ≥ 46 g, 2.22 (1.47–3.36), respectively] (Table 3). After adjusting for clinically relevant factors, ≥ 46 g of alcohol consumption was also identified as a significant predictor of the incidence of low eGFR [non-drinker, 1.00 (reference); < 23 g, 1.23 (0.91–1.65); 23–46 g, 1.21 (0.83–1.79); and ≥ 46 g, 1.62 (1.04–2.53), respectively] (model 3, Table 3). Daily alcohol consumption (per g/day) was not linearly associated with the outcome; hazard ratios (95% confidence interval): 1.01 (0.99–1.01).

We performed a multiple imputation analysis as a sensitivity analysis for missing data regarding alcohol consumption and clinical outcomes. The results of this analysis were similar to those of the original analysis (Additional files 1 and 2).

## Discussion

The present cohort study, which included 26,788 members of the general population with non-CKD, revealed that a daily higher alcohol consumption (≥ 46 g/day) was significantly associated with a higher incidence of proteinuria and low eGFR among women. These findings suggest that women might be vulnerable to excess alcohol consumption, which might lead to kidney dysfunction. An advantage of the present study is that it confirmed the robustness of the influence of alcohol consumption on two clinically important outcome measures of kidney dysfunction (incidence of proteinuria and eGFR < 60 mL/min/1.73 m^2^ with 25% decline).

Although multiple observational cohort studies have evaluated the relationship between alcohol consumption and incidence of CKD [30], incidence of proteinuria [11, 17, 23, 31], incidence of low eGFR [10, 11, 13, 18], and GFR annual decline [22], these results were not entirely consistent, possibly due to differences in sample sizes, definitions of alcohol intake levels and CKD outcome measures, or participant lifestyles.

Among several large cohort studies that evaluated the relationship between alcohol consumption and incidence of proteinuria, Yamagata et al. reported that an average daily alcohol consumption of ≤ 20 g of ethanol was associated with a decreased risk of future proteinuria in Japanese men and women compared with non-drinkers, while consumption of > 20 g of ethanol was not associated with the risk of future proteinuria [23]. However, this study did not evaluate the clinical impact of heavy alcohol consumption on the incidence of proteinuria. Uehara et al. showed that high alcohol consumption (> 69 g/day) [32], had a significant J-shaped association with proteinuria incidence in 9154 non-diabetic Japanese men. However, this study did not include women; thus, the effects of high alcohol consumption in women remained unknown. Recently, Kimura et al. demonstrated that women were more vulnerable to high doses of alcohol consumption (≥ 60 g/day) than men in terms of proteinuria incidence in a general population-based large retrospective cohort study that included 11,286 Japanese male and female participants [17]. Although this previous study was compatible with the present study, the previous study only focused on the incidence of proteinuria as a surrogate marker of ESRD; it should be evaluated by other clinically important surrogate renal outcomes, such as eGFR.

Meanwhile, among the other few studies that evaluated both incidence of proteinuria and eGFR decline as a CKD surrogate marker, one prospective cohort study that included 6259 Australian male and female participants showed that the risk of developing new-onset albuminuria rose for an average daily alcohol consumption (≥ 30 g/day), compared to that with consumption of < 10 g/day [11]. However, inversely, the risk of developing a low eGFR was significantly reduced at the same threshold. This discrepancy should be cautiously interpreted. In addition, another recent large, nationwide, retrospective cohort study conducted in Korea, including 118,492 participants, assessed the relationship between alcohol consumption and the incidence of CKD, defined as the incidence of proteinuria, low eGFR (eGFR < 60 mL/min/1.73 m^2^), and eGFR annual decline [22]. The results of this study revealed a J-shaped association between alcohol consumption and incident proteinuria in men, with men consuming < 10 g of alcohol/day having a lower risk of proteinuria development that that of men consuming higher levels of alcohol (≥ 40 g/day), compared to that in non-drinkers. A positive association was observed between alcohol consumption and proteinuria in women with any range of consumption compared with that in non-drinkers. However, when evaluating the low eGFR incidence and eGFR annual decline as outcomes, a negative association between alcohol intake and outcomes was observed in both sexes. These results were conflicting in each CKD outcome definition; therefore, these results should be interpreted cautiously. Meanwhile, in another large, prospective, population-based cohort, including 5476 male and female participants in the Netherlands, alcohol consumption was inversely associated with the risk of developing CKD, defined as either an eGFR < 60 mL/min/1.73 m^2^ or proteinuria (24-h urinary albumin excretion > 30 mg) [12].

In the present study, we clarified that a larger amount of alcohol consumption was a risk factor for the incidence of proteinuria and low eGFR in women. The findings of the present study, as well as those of a previous study [17], suggest that avoiding excess alcohol consumption might be an important lifestyle modification to prevent kidney dysfunction, especially in women.

Although the precise mechanism of the nephrotoxic effect of alcohol remains unelucidated [32], one of the plausible mechanisms by which excess alcohol consumption induces kidney dysfunction is considered to be by inducing the depression of nephrin and podocin in podocytes, which causes proteinuria, leading to kidney dysfunction, which is mediated by oxidative stress [33]. Furthermore, previous studies have shown that higher alcohol consumption is associated with an increased risk of future hypertension [34], leading to the incidence of proteinuria [26]. In the present study, the difference in blood pressure between each alcohol consumption group was not clinically significant, suggesting that the difference in blood pressure between each alcohol consumption group did not influence the occurrence of kidney dysfunction. Conversely, another study suggested that alcohol consumption might improve kidney antioxidant activities and capacity; namely, a small amount of ethanol pretreatment can increase the activities of inducible nitric oxide synthase and antioxidant capacities in the kidneys, which ameliorated oxidative stress in a bilateral renal ischemia reperfusion simulation model as a compensatory mechanism [35]. However, the optimal range of alcohol consumption to induce a positive effect on kidney function is unknown. Thus, further studies should evaluate this issue.

Regarding the mechanism for the sex difference in the impact of alcohol on kidney function, one possibility may be the different pharmacokinetics of alcohol between males and females. Compared with males, females are more likely to have higher concentrations of alcohol, partly because females, with a lower proportion of body water, have a smaller distribution volume of alcohol [17, 36]. Furthermore, the sex difference in alcohol impact may be attributed to the differences in alcohol metabolism because of the lower activity of alcohol dehydrogenase in females than in males, leading to a higher concentration of alcohol in women than in men, even within a similar level of alcohol consumption [35]. Further studies should be conducted to explain these mechanisms.

This study has several limitations. First, self-reported alcohol consumption may be biased. Second, in the present study, proteinuria was measured using a dipstick, but dipstick tests are more likely to yield false-positive and false-negative results than specific laboratory methods. Third, because of the retrospective nature of the present study, confounding factors such as excess alcohol consumption, unhealthy behaviors, and calorie-dense and hypersaline foods could not be evaluated [37]. Fourth, herein, we had no data regarding death, income level, or education, which may be related to alcohol consumption; therefore, further studies should include these data during evaluation.

## Conclusions

This general population-based retrospective cohort study revealed that daily higher alcohol consumption was significantly associated with a higher incidence of proteinuria and an eGFR < 60 mL/min/1.73 m^2^ with a 25% decline in eGFR among women. Based on our results, high alcohol consumption is a modifiable lifestyle-related factor to prevent CKD in women. Women might be prone to high alcohol consumption with kidney dysfunction.

## Supplementary Information

**Additional file 1.** Alcohol consumption and the incidence of proteinuria in males and females [table].

**Additional file 2.** Alcohol consumption and the incidence of low eGFR in males and females [table].

## Data Availability

All data generated or analyzed during this study are included in this published article.

## Abbreviations

BMI: Body mass index
CKD: Chronic kidney disease
eGFR: Estimated glomerular filtration rate
ESRD: End-stage renal disease
