# Supplementary material for: Association of alcohol consumption with the incidence of proteinuria and chronic kidney disease: a retrospective cohort study in Japan
Source: Nutr J. 2022 May 14;21:31. doi: 10.1186/s12937-022-00785-x (PMC9107250; doi:10.1186/s12937-022-00785-x)
Supplement: Supplementary file 2 — Additional file 2. Alcohol consumption and the incidence of low eGFR in males and females [table]. [file 12937_2022_785_MOESM2_ESM.docx]

**Additional file 2**

**Alcohol consumption and the incidence of low eGFR in males and females**

|  | **Daily alcohol consumption categories (g of alcohol)** | | | |
| --- | --- | --- | --- | --- |
|  | **No** | **<23** | **23–46** | **≥46** |
| **Males** |  |  |  |  |
| Adjusted model | 1.0 (reference) | 1.02 (0.84–1.23) | 0.90 (0.72–1.11) | 0.92 (0.70–1.21) |
| **Females** |  |  |  |  |
| Adjusted model | 1.0 (reference) | 1.22 (0.91–1.64) | 1.21 (0.82–1.78) | 1.60 (1.03–2.49) * |

PY, person-years; CI, confidence interval.

Multivariable model by multiple imputation analysis for missing data: adjusted for age (years) and eGFR (mL/min/1.73 m^2^) at baseline, body mass index (BMI) (kg/m^2^), smoking status (never/former and current smokers), and current treatment for comorbidities (hypertension, dyslipidemia, diabetes mellitus, and cardiovascular disease).

**P* < .05.
